# Dangerous Ground: One-Year-Old Infants are Sensitive to Peril in Other Agents’ Action Plans

**DOI:** 10.1162/opmi_a_00063

**Published:** 2022-10-30

**Authors:** Shari Liu, Bill Pepe, Manasa Ganesh Kumar, Tomer D. Ullman, Joshua B. Tenenbaum, Elizabeth S. Spelke

**Affiliations:** Department of Brain and Cognitive Sciences, MIT; Department of Psychological and Brain Sciences, Johns Hopkins University; Department of Psychology, University of California San Diego; Department of Psychology, University of Bath; Department of Psychology, Harvard University; Center for Brains, Minds and Machines, MIT

**Keywords:** cognitive development, infancy, agency, action understanding, open data, open materials, pre-registered

## Abstract

Do infants appreciate that other people’s actions may fail, and that these failures endow risky actions with varying degrees of negative utility (i.e., danger)? Three experiments, including a pre-registered replication, addressed this question by presenting 12- to 15-month-old infants (*N* = 104, 52 female, majority White) with an animated agent who jumped over trenches of varying depth towards its goals. Infants expected the agent to minimize the danger of its actions, and they learned which goal the agent preferred by observing how much danger it risked to reach each goal, even though the agent’s actions were physically identical and never failed. When we tested younger, 10-month-old infants (*N* = 102, 52 female, majority White) in a fourth experiment, they did not succeed consistently on the same tasks. These findings provide evidence that one-year-old infants use the height that other agents could fall from in order to explain and predict those agents’ actions.

## INTRODUCTION

*El Capitan* is a wall of sheer granite rising 3000 feet from the ground in Yosemite Park, and scaling it takes effort. But scaling *El Capitan* without safety gear is not just effortful; it is fraught with danger. Danger describes the properties of a situation, but it also tells us something about people’s actions, values, and reasoning. If we see a parent chasing a child who is running along a dangerous cliff, we may recognize that the child is unaware of the danger, and that the parent is willing to accept that danger to themselves because of the high value of bringing their child to safety. These examples are extreme, but predicting and explaining other people’s actions are part of our everyday lives: We consider both concrete action costs (e.g., physical effort) and costs abstracted away from actions themselves (e.g., opportunity costs, risks, and perils), and we reason not only about what has happened, but also what could have happened, and what could happen next. How and when does such reasoning develop? Here, we explore the early development of these abilities, when infants observe an agent that engages in dangerous but consistently successful actions.

Our ability to use observations of others’ behavior to reason about their hidden mental lives (often termed *intuitive psychology*) has been a focus of cognitive science for more than half a century (Dennett, 1987; Heider & Simmel, 1944; Perner, 1991; Premack & Woodruff, 1978; Wellman, 2002). Adults, children, and infants are sensitive to physical constraints on other agents’ actions and expect others to act efficiently by minimizing effort (Baker et al., 2009, 2017; Gergely & Csibra, 2003; Jara-Ettinger et al., 2015, 2016; Liu et al., 2017; Liu & Spelke, 2017). In one series of experiments testing for sensitivity to physical cost in human infants, participants first see movies of animated agents or real people move or reach over a barrier towards a goal. Then, infants see test events in which the intervening obstacle is removed. In one event, the agent moves in the same curved path as they did before, but the absence of the obstacle makes this familiar path appear inefficient. In the other event, the agent moves efficiently, on a direct but novel path to the goal. The main finding from this literature is that infants look longer at the inefficient action at test, even though the action itself is familiar, than at the efficient action, even though the action itself is novel. From these experiments and many control conditions, researchers infer that infants expect others to act efficiently with respect to their goals (Gergely & Csibra, 2003).

**Figure 1.**
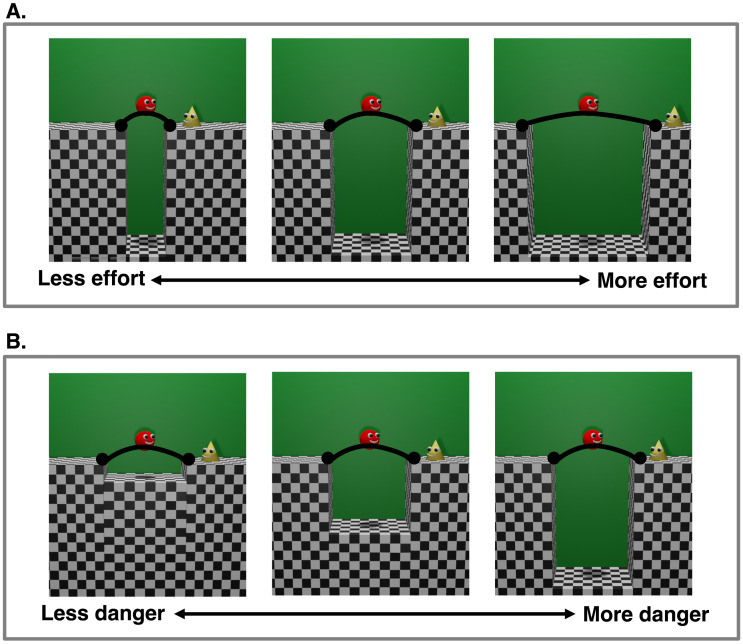
**Actions used in (A) previous research, manipulating physical action cost, and in (B) the present research, manipulating danger.** (A) As the width of the trench increases, all else being equal, successful jumps follow a longer path and are perceived by adult observers to require greater force, be more exhausting, and be less likely to succeed (see SM). (B) As the depth of the trench increases, all else being equal, successful jumps remain physically identical but are perceived by adult observers to entail greater danger (Gjata et al., 2022).

Moreover, infants use the physical costs of the actions that an agent took to attain its goals to infer the value of these goal states to the agent (Liu et al., 2017). In these experiments, infants first saw an agent take a low-cost action and reject a medium-cost action to arrive at one goal, and accept a medium-cost action and reject a high-cost action to arrive at the other goal, where the cost of the action varied with the height of an intervening barrier, the angle of an inclined ramp, or the width of a gap in the surface of support (Figure 1). The experiments tested whether infants would infer that the agent preferred the goal it worked harder to reach, solely on the basis of how much cost they saw the agent incur. To investigate this question, infants saw test events in which the agent was situated equidistant from the two goals, and chose each of them in turn. The main finding was that infants looked longer when the agent had performed more costly actions for one goal but then chose to approach the other goal in the test events. Because infants responded similarly to actions with varying path lengths, vertical movement, horizontal movement, and acceleration profiles across experiments, no single perceptual variable accounts for their responses. Rather, infants appear to represent all of these actions as converging on a single variable: physical cost.

### Beyond Physical Effort: Perception of Danger

Actions can carry negative utility for reasons beyond physical effort: They can require mental effort, have a low probability of success, or lead to bad outcomes if the actor fails to complete them. For example, consider the trenches depicted in Figure 1, inspired by a vast literature on depth perception and motor development in humans and other animals (Adolph, 2000; Adolph & Kretch, 2012; Gibson & Walk, 1960; Lashley & Russell, 1934; Walk et al., 1957). The action of jumping across wider versus narrower trenches (Figure 1A) differ both in physical effort (wider trenches require longer, more forceful jumps) and probability of failure (jumps over wider trenches are more likely to fail), but the consequences of failure are roughly equal, because the agent falls from the same height in all cases. In contrast, physically identical jumps across both deeper and shallower trenches (Figure 1B) follow the same path, require equal effort, and carry an equal probability of failure. But, the negative consequences of falling into a deep trench are worse than following into a shallow one—the deeper the trench, the farther the fall, and the greater the injury. Such intuitions are verified empirically. We collected a pilot dataset on adult participants (*N* = 19 from Amazon Mechanical Turk) gauging these intuitions, and found that varying the width of the trench strongly modulated people’s intuitions about the probability of success, the force required to jump, and the perceived effort of the jump, more so than manipulations of depth. See Supplemental Materials for full results, and Gjata et al. (2022) for evidence that manipulations of trench depth influence how adults and children predict and explain other agents’ actions.

Where does our ability to perceive the danger behind other people’s actions come from? Past studies show that with motor experience, human infants and toddlers begin to avoid stepping into or reaching across the edge of a sheer drop-off (Gibson & Walk, 1960; Kretch & Adolph, 2013), and are less willing to cross over deeper trenches (Kretch & Adolph, 2013). However, it is unclear how infants would respond to this manipulation in the context of other agents’ actions, when demands on their own motor planning are removed. To test whether infants are sensitive to the dangers underlying other people’s actions, we used the experimental logic of the prior studies of Gergely et al. (1995) and Liu et al. (2017), and stimuli adapted from the classic visual cliff literature and Gjata et al. (2022), in which an agent performed identical jumps across the trenches shown in Figure 1B. Importantly, the agent always jumped successfully to its goal, and all properties of its action were the same across the events. By varying the depth of the trenches, we manipulated the negative utility (i.e., ‘danger’) that would have resulted had the action failed. In order to appreciate these actions as dangerous, therefore, infants must look beyond the physical actions taken by the agent and analyze the surrounding physical context in which the agent acts. Experiment 1 tested whether infants use the degree of danger of an action to infer the value of its goal for the actor. Experiment 2 tested whether infants expect others to minimize the danger of their actions. Experiment 3 was a replication of Experiment 2 that tested whether infants would still have the same expectations if they did not have the opportunity to associate deeper trenches with more physical damage during the study. In Experiment 4, we tested younger infants using methods identical to those in Experiments 1–3. Overall, we found that infants older than one year of age learned what other agents prefer from observing the degree of danger these agents withstood, and expected agents to minimize danger. By contrast, infants under one year of age did not succeed consistently in either of these tasks.

## EXPERIMENT 1

Experiment 1 used the methods of our past research on infants’ inferences of goal values from physical costs (Liu et al., 2017). We tested whether 13-month-old infants infer the value of a goal to an agent from the danger of the action that the agent undertook to obtain it, by varying how far an agent would fall if its action failed, holding constant all physical properties of the agent’s movement.

### Methods

#### Participants.

Our final sample of participants included 32 thirteen-month-old infants (*M* = 12.9 months, range = 12.6–13.5, 17 female). Seven infants were excluded and replaced due to fussiness (3 infants) or inattentiveness during test trials (4 infants). Participants were recruited through a database of families who expressed interest in cognitive development research in the Greater Boston Area. Of the families in this database who chose to provide demographic information, 79.5% identified their children as White, 10.2% as Asian, 6.9% as Other, 2.5% as Black or African American, 0.4% as American Indian/Alaska Native, and 0.4% as Native Hawaiian/Pacific Islander; 90.3% as not Hispanic or Latino, 9.5% as Hispanic or Latino, and 0.2% as both. Most families in the database (90.4%) had at least one parent or legal guardian with a college diploma or higher. All data were collected at the Harvard Lab for Developmental Studies with procedures approved by the Harvard University Committee on the Use of Human Subjects. We studied 13-month-old infants, rather than 10-month-old infants that participated in previous studies of similar design (Liu et al., 2017) because the younger infants lack experiences with walking and falling that may foster the development of these abilities. The sample size was chosen based on a simulation power analysis over the confirmatory analyses from 2 previous experiments with similar structure, conducted with 10-month-old infants (Experiments 1–2 from Liu et al., 2017), and we collected data until we attained our pre-specified N. The full pre-registration document, including details about methods, sample size, hypotheses, and analysis plan, along with original data, scripts, and stimuli, are available at https://osf.io/kz7br/.

#### Displays.

**Figure 2.**
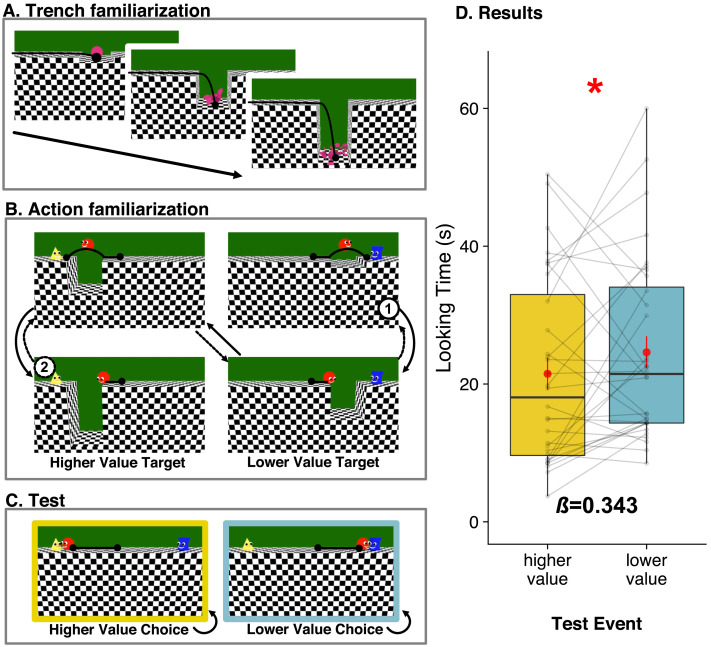
**Trial and event structure (A–C) and main results (D) of Experiment 1.** Infants first saw (A) a video of an inanimate object falling off the edge of a shallow, medium, and deep trench, and breaking. Then, during action familiarization (B), infants saw the agent jump across a shallow but not a medium trench for one goal (Blue, right panels) and a medium but not a deep trench for the other goal (Yellow, left panels). All jumps and all refusals were depicted with physically identical movements except for their location and direction, which were counterbalanced. Each familiarization trial included all 4 events played in a loop in two orders. In order 1, the agent jumped over a shallow trench on one side, refused to jump over a medium trench on that side, jumped over a medium trench on the opposite side, and refused to jump over a deep trench on that side. In order 2, the order of these four actions was reversed Each order looped and repeated, contingent on infants’ looking time; the two orders appeared on alternating trials. Finally, at test (C), infants saw the agent face a choice between the two goals from familiarization, and alternatingly chose to approach each one. Black lines within each still image indicate trajectories of motion, and filled circles indicate start- and end-points of motion. Across infants, the order of the first familiarization trial (1 or 2) and the direction and target of the more dangerous action were counterbalanced. (D) Looking time in seconds towards the test events in Experiment 1 (*N* = 32 13-month-old infants). Red error bars around means indicate within-subjects 95% confidence intervals. Pairs of points indicate data from a single participant. Horizontal bars within boxes indicate medians, and boxes indicate the middle 2 quartiles of data. Beta coefficients (*β*) indicate effect sizes in standard deviations. * Indicates pre-registered *p* < .05, two-tailed.

Animated videos were created in Blender (Blender Development Team, 2021). All stimuli were projected to a 1.02 × 1.32 m screen and displayed using Keynote. The actions took place on a 3D surface layout with a uniform checkerboard texture, whose depth and structure were specified by gradients of texture density, as in Eleanor Gibson’s classic studies on the visual cliff (Gibson & Walk, 1960). Infants and non-human animals use these depth and surface cues to perceive visible surfaces of support and the relative positions of surfaces that vary in distance and depth, as early as 7 months of age (Kavšek & Granrud, 2013; Walk et al., 1957; Yonas et al., 1986). The *trench familiarization trials* (Figure 2A) presented a ball emerging from one side of the screen (side counterbalanced across participants) and rolling into a shallow (1 unit in Blender space), medium (8 units), and deep (15 units) trench at the center of the screen (7.0s each, with a 0.5 black screen after), in that order, and falling to the bottom. Depending on the depth of the trench, the ball remained unbroken, broke into 5 pieces, or shattered into 100 pieces, with louder and longer sounds of breaking and shattering paired with longer falls.

For the *action familiarization trials* (Figure 2B), infants saw 4 distinct animated events, played on a loop in two orders. For each event, the red agent began at the center of the screen and one of the targets appeared to its left or right, beyond a shallow, medium, or deep trench. The target jumped up and down twice, making a noise, the agent turned and moved to the edge of the trench, and either jumped over it (making a positive “Mmmm!” sound) (9.4 s), or declined, backing away from its edge (making a reluctant “Hmmm …” sound) (7.7 s), followed by a 0.5 s black screen before the next action. On each trial, these 4 events were played in one of two orders: from low to high trench depth (order 1 in Figure 2B), or the reverse (order 2 in Figure 2B). The location of the deeper trench (left vs. right) and more valued goal (blue vs. yellow), and the order of the first familiarization trial (1 or 2), were counterbalanced across participants. Thus, infants saw the agent accept and refuse equally effortful actions towards each target with equal frequency and affect. Indeed, all the actions were physically identical except for their direction (left vs. right, counterbalanced across infants). Relative trench depth (hence, degree of danger) was the only variable that distinguished actions toward the two targets.

In the *test events* (Figure 2C), which also looped, all three characters were presented in the same scene, in the same locations as in familiarization, with no intervening trenches. The central agent looked between the targets, making an uncertain ‘Hmmmm …’ sound (2.5 s), and then moved on alternating trials to the target for which it had jumped deeper trenches (hereafter, the higher value goal), and to the other target (the lower value goal) (3.4 s per action).

#### Procedure.

Infants were seated on their caregiver’s lap, approximately 1.5 m from the projector screen. Prior to the experiment, an experimenter attracted the baby’s attention to the left, right, top, and bottom edges of the screen, generating calibration images to guide human coders. Caregivers were asked to keep their eyes closed and to refrain from interacting with their infants during the experiment. All infants saw 1 trench familiarization trial (Figure 2A), 6 action familiarization trials (Figure 2B), and 2 pairs of test trials (Figure 2C). Other than trench familiarization, which was fixed in length, all trial durations depended on infants’ looking times to the displays, and ended after infants looked at the events for 60 seconds total, or looked away from the screen for 2 consecutive seconds (see Figure SS2 for infants’ looking times during the familiarization trials, and the SM for an analysis of infants’ attention during each of the 4 video clips from action familiarization trials).

#### Data Coding and Analysis Strategy.

Infant looking times were coded online using XHAB (Pinto, 1995), and offline using Datavyu (Datavyu Team, 2014). All experimenters and coders were naive to the order of the test events and unable to see the video events (they relied on sound cues to start each trial). To check for exclusions and coding errors, all test trial data were re-coded in Datavyu and excluded if an infant looked away from a test event without ever having seen the agent jump, or if the trial ended too early or late (15 out of 320 total familiarization trials). We used these offline coded looking times for our final analyses. To assess the reliability of the data, (160 out of 320 trials) were re-coded in Datavyu by an additional researcher who was naive to test event order. Reliability was high, intraclass correlation coefficient (*ICC*) = 0.97, 95% confidence interval = [0.95, 0.98]. All decisions to include or exclude trials or participants from our analysis were made by researchers who did not know the order of events shown to infants.

Infant looking times often are log-normally distributed (Csibra et al., 2016), including in this dataset (log-likelihood of average looking times during test and control trials for Experiments 1–4 under normal distribution −2624.45, under lognormal distribution = −2456.77). Our pre-registered dependent measure therefore was the average looking time towards the higher- or lower-danger choice at test in log seconds. All analyses were conducted on this measure, but our summary statistics and plots feature untransformed looking times for interpretability. We analyzed all looking times using mixed effects models (Bates et al., 2015) using Satterthwaite’s degree of freedom method, implemented in R (R Core Team, 2020). Analyses with repeated measures included a random intercept for participant identity; those conducted over multiple experiments included a random intercept for experiment. For every model, we checked for influential participants using Cook’s Distance (Nieuwenhuis et al., 2012) and excluded participants who exceeded the standard 4/*n* threshold, where *n* is the number of participants. The number of participants who met this criterion is listed in every model result; including or excluding them does not change the interpretation of any primary analysis (for results including all observations, see Supplemental Materials). Data manipulation and plotting were conducted using the tidyverse packages (Wickham et al., 2019). Cohen’s D derived from lme models were calculated using the EMAtools package (Kleiman, 2017). To enhance reproducibility, all results were written in R Markdown (Xie et al., 2018).

Prior to conducting Experiment 1, we did not have a clear prediction for the direction of infants’ looking preferences during test events and therefore pre-registered a two-tailed alpha of .05 for that experiment. In light of the findings of Experiment 1, we predicted longer looking to unexpected events across Experiments 2–3 and therefore pre-registered a one-tailed alpha threshold of .05 for infants’ looking preferences during the test events. We therefore report one-tailed significance values for analyses of all the effects that replicate or extend those of Experiment 1, and two-tailed p-values for all other analyses.

#### Alternative Hypotheses.

If infants are sensitive to danger in other people’s actions and view potential falls from greater heights as more negative even when the actions are successful and their motions are identical, then infants should infer that people who take more dangerous actions towards certain goals value those goals more highly, as they do for people who take more effortful actions toward those goals (Liu et al., 2017). Under this hypothesis, we expected infants’ looking time during the test trials to differ, depending on whether they saw the agent choose the goal for which it jumped deeper versus shallower trenches. Alternatively, if infants are sensitive to the physical effort of actions based on their paths, their profiles of velocity or acceleration, or their manner, but cannot appreciate that two physically identical actions can nonetheless differ in danger, then they should attribute approximately equal value to the two goals and look equally at the two test events.

### Results

Infants looked longer when the agent chose the target achieved through the less dangerous action (*M*_*lowervalue*_ = 24.60 s, pooled standard error (*SE*) = 1.14), than when the agent chose the target achieved through the more dangerous action (*M*_*highervalue*_ = 21.51 s, *SE* = 1.14). See Figure 2D. This difference was significant (95% confidence interval (CI) over difference in log seconds [0.02, 0.39], standardized beta coefficient (*β*) = 0.34, unstandardized coefficient (*B*) = 0.212, t value over degrees of freedom *t*(31) = 2.16, *p* value (*p*) = 0.039, two-tailed, Cohen’s *d* = 0.79, no influential observations). As in the experiments of Liu et al. (2017) using similar methods, but presenting physically different actions on the two test trials, infants looked longer when the central agent performed costlier (previous work) or more dangerous (current work) actions for one goal than another, and then chose against this goal at test. See Figures S3–4 and associated supplemental text for evidence against an alternative interpretation of these results, according to which infants attended to and compared only the jumps over trenches of medium depth without using the relative differences in trench depth across the presented actions.

## EXPERIMENT 2

Experiment 1 provided evidence that infants inferred the relative value of two goals to an agent given the amount of danger the agent withstood for each of them, even though the agent’s actions always succeeded and were physically identical. Experiment 2 used a different task to ask whether one-year-old infants expect others to minimize the danger of the actions that they direct to two targets of approximately equal value, using the logic of previous studies (Gergely et al., 1995; Gjata et al., 2022; Liu et al., 2017; Liu & Spelke, 2017).

### Methods

This study was originally pre-registered with a sample including both 10-month-old and 13-month-old infants. For clarity of presentation, we report the findings from 13-month-old infants first, and then present the findings from 10-month-old infants along with other studies of the same age group in Experiment 4. Our pre-registration document, data, and stimuli are available at https://osf.io/kz7br/.

#### Participants.

Our final sample of participants included 30 thirteen-month-old infants (*M* = 12.89 months, range = 12.53–13.50, 12 female). We chose this sample size using a simulation power analysis over the confirmatory analysis of data from a pilot study, as well as estimates of effect sizes of studies with similar displays and design (Liu et al., 2017). We collected data until we attained our pre-specified N. Infants were excluded and replaced in the final sample due to fussiness that prevented study completion (3 infants), inattentiveness during test trials (2 infants), or interference from caregivers (2 infants).

#### Materials, Design, and Procedure.

**Figure 3.**
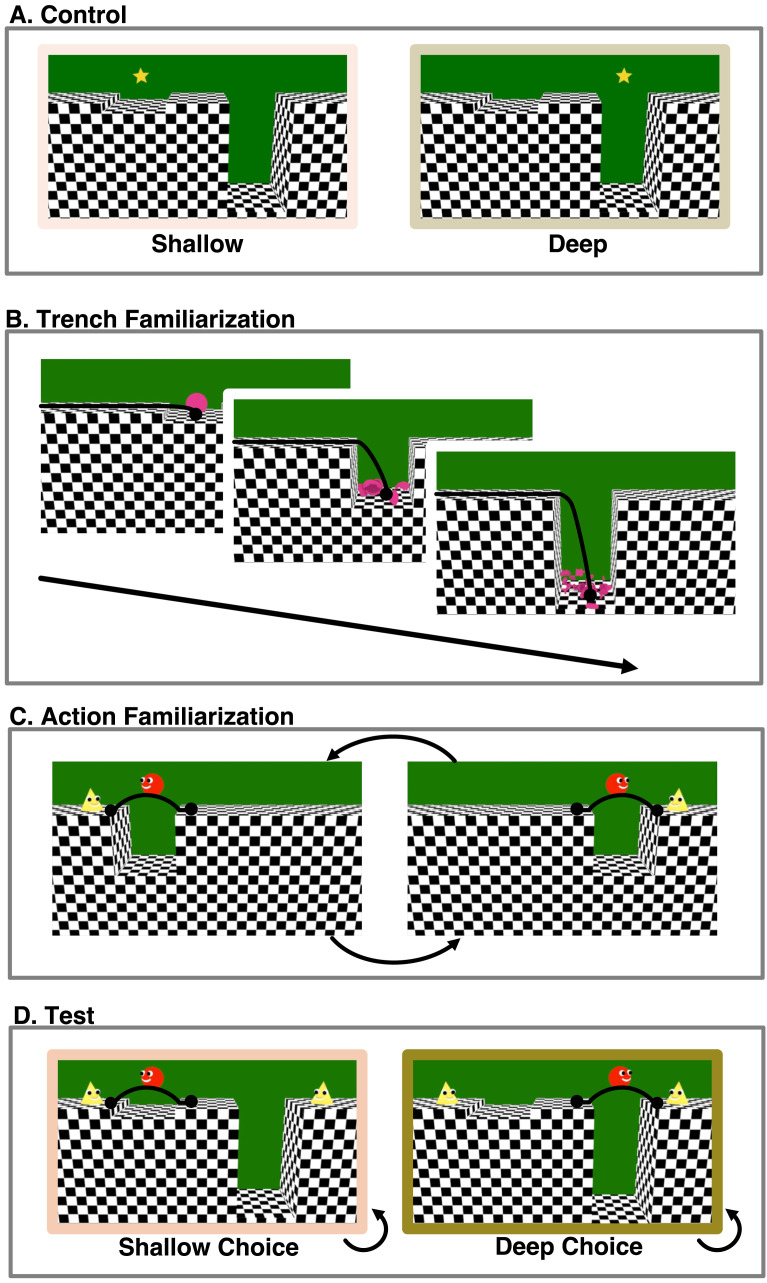
**Trial and event structure of Experiment 2.** Infants first saw (A) an attention-getting star wiggle above the deep and shallow trench. Next, they saw (B) a video of an inanimate object falling off the edge of a shallow, medium, and deep trench and breaking not at all, into 5 pieces, or into 100 pieces respectively. Infants then saw (C) the agent jump over trenches of slightly varying medium depth (9, 10, and 11 Blender units - 10 units pictured here) to two target characters of identical appearance, standing in alternation on the left and right sides of the display. Finally, infants saw (D) the agent facing a choice between the two targets, one beyond a shallow trench (2 Blender units) and the other beyond a deep trench (18 Blender units), and alternately choosing to jump over to each target. Black lines within each still image indicate trajectories of motion, and circles indicate start- and end-points of motion. All jumps were physically identical except for the location and direction of movement, which were counterbalanced. Black arrows outside of each still image indicate presentation order (B–C), and/or looping (C–D). Across infants, the order of the actions and the direction and target of the more dangerous action were counterbalanced.

Figure 3 depicts the materials and procedure; infants saw 2 control trials, 1 trench familiarization trial, 6 action familiarization trials, and 2 pairs of test trials. As in Experiment 1, familiarization, test, and control trials played on a loop and ended after infants looked for 60 s total or looked away for 2 s consecutively.

To assess infants’ interest in the deep and shallow trenches outside of the context of goal-directed action, two *control* trials (Figure 3A) presented a shallow and a deep trench with no animated characters. An attention-getting star appeared above each of the trenches (order counterbalanced across participants) accompanied by a sound. Next came a *trench familiarization* trial (Figure 3B) identical to that of Experiment 1, followed by 6 *action familiarization* trials (8.9 s apiece with a 0.5 s black screen following each event; Figure 3C), played in a continuous, sequential loop, wherein the agent performed identical jumps over trenches of 3 intermediate depths towards two target agents. Each familiarization trial included all 6 of these events, played in one of two pseudo-random orders that counterbalanced the starting position (left or right) of the target and trench. In each event, the target jumped up and down twice, making a noise, the agent turned to look in its direction, moved towards the trench, looked down, and backed up and jumped over it, landing next to the target, making the same positive “Mmmmm!” vocalization as in Experiment 1. There are two plausible ways to interpret these stimuli. First, using the logic of Experiment 1 and its predecessors (Liu et al., 2017), the observer could infer that the two targets are of approximately equal value–the agent was willing to leap over trenches of equal depth to reach each of them. Second, the observer could see these actions as directed towards the same agent that sometimes appeared on the left and sometimes on the right side of the screen. Under either interpretation, infants see that the agent leaps towards the right versus the left, over trenches of medium depth, with equal frequency.

To test whether infants expect agents to choose less over more dangerous actions, we then presented 4 test trials (Figure 3C), in which the central agent chose to jump either a shallow or deep trench, when both were presented simultaneously. Like in Experiment 1, these test trials presented the three characters in the same scene, but unlike Experiment 1, reaching either of the two targets required the central agent to jump over either a shallow or a deep trench. After the two targets jumped up and down twice, the agent turned towards each target, making the same uncertain “Mmmmm …” sound as in Experiment 1, and then jumped across the deeper or shallower trench on alternating looped trials, following the same action path as during familiarization (7.2 s per event, with a 0.5 s black screen following each event). Across infants, we counterbalanced the order of events in the first familiarization trial (though all infants saw 3 trials of each kind, presented in alternating order), the order of the shallow and deep jumps in the test events and in the control events, and the side of the deeper trench.

#### Data Coding and Analysis.

The data coding and analysis strategies were the same as in Experiment 1. Twenty-five out of 360 control and test trials were excluded from the analysis based on inattentiveness or coding error. Half the test trials from the experiment (60/120 trials) were re-coded in Datavyu by an additional researcher who was naive to test event order. Reliability was high, *ICC* = 0.99, 95% *CI* [0.999, 0.999].

#### Alternative Hypotheses.

Under the hypothesis that infants expect others to choose less dangerous over more dangerous (but otherwise identical) actions, infants will look longer when the agent jumps over the deep trench. Under the alternative hypothesis that they do not see jumps over deeper trenches as more dangerous and therefore more costly, they will look equally to the two test events, which show two physically identical actions.

### Results

**Figure 4.**
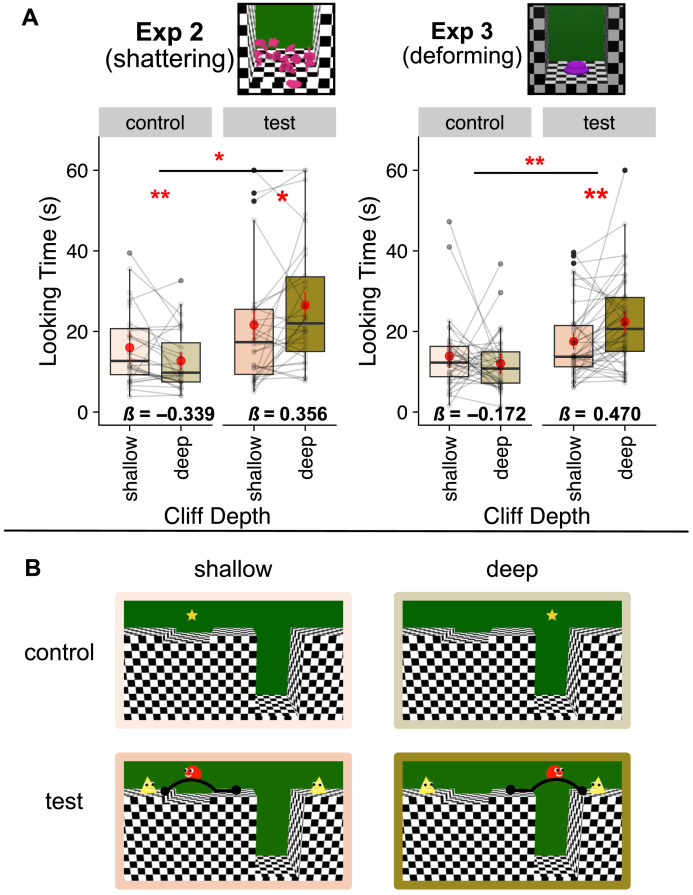
**(A) Looking response to test and control events and (B) example stimuli in Experiment 2 (*N* = 30 13-month-old infants) and Experiment 3 (*N* = 42 12 to 15-month-infants).** Boxplots indicate data from the test events, in which the agent chose to jump across a deeper or shallower trench for a goal, and the control events, in which a star drew infants’ attention to the deeper and shallower trenches. Red error bars around means indicate within-subjects 95% confidence intervals. Pairs of points indicate data from a single participant. Horizontal bars within boxes indicate medians, and boxes indicate the middle 2 quartiles of data. Beta coefficients (*β*) list effect sizes in standard deviations. *P* * < .05, ** <.01, *** <.001 (pre-registered as one-tailed for test events due to directional prediction, two-tailed in all other cases). (B) Still frames from the control and test events across the test and control events.

Infants looked longer when the agent, at test, chose to cross the deeper over the shallower trench (*M*_*deep*_ = 26.5 s, *SE* = 1.61; *M*_*shallow*_ = 21.64 s, *SE* = 1.95; [0.03, 0.43], *β* = 0.36, *t*(28) = 2.33, *p* = .014, one-tailed, *d* = 0.88, excluding one influential participant). In contrast, when infants’ attention was drawn to each trench by an attention-getting star that hovered above it, infants looked longer at events near the shallow trench (*M*_*deep*_ = 12.73 s, *SE* = 1.11; *M*_*shallow*_ = 16.02 s, *SE* = 2.00; [−0.31, −0.08], *β* = −0.34, *t*(25.1) = −3.24, *p* = .003, two-tailed, *d* = −1.29, excluding 2 influential participants). Looking preferences between the control and test events differed significantly ([0.11, 0.88], *β* = 0.75, *t*(84.74) = 2.52, *p* = .013, two-tailed, *d* = 0.55, no influential observations). See Figure 4A.

### Discussion

Together, Experiments 1 and 2 provided evidence that 13-month-old infants expect other agents to choose safer over more dangerous actions, and they infer that an agent’s willingness to tolerate greater danger to reach one goal is motivated by the higher reward of that goal for the agent. However, the source of this expectation is not fully clear, because the initial familiarization event presented an inanimate object that landed intact in the shallow trench and broke when it landed in the medium or deep trench–state changes that adults perceive as negative. Do infants come into the experiment appreciating that deeper trenches are more dangerous, or did infants associate the deeper trench with the sound and sight of an object breaking, interpret this outcome as negative, and generalize this association to all subsequent events presented in the experiment? We address this question in Experiment 3.

## EXPERIMENT 3

Experiment 3 tested whether infants expect an agent to minimize the danger of its jumps over trenches of varying depths, removing evidence that inanimate objects undergo enduring damage when dropped into deeper trenches.

### Methods

#### Participants.

Our final sample included 42 twelve- to fifteen-month-old infants (*M* = 13.95 months, range = 12.29–15.67, 24 female): a slightly wider age range that enabled more rapid testing of participants, who were recruited both from our lab database, and also through a cross-institution platform for recruitment for developmental cognitive science (https://childrenhelpingscience.com/). Our preregistered target sample size of 40 was determined based on a simulation power analysis over infants’ looking preferences towards the test events from Experiment 2; our stopping rule was to stop recruiting as soon as we reached our target N, but to finish collecting data if we over-recruited. Thus, our final sample was *N* = 42. A further 6 infants were excluded from the study (3 due to technical issues, 2 due to inattentiveness and 1 due to interference from the caregiver). Our pre-registration, original data and scripts, and stimuli are available at https://osf.io/kz7br/.

#### Materials and Design.

**Figure 5.**
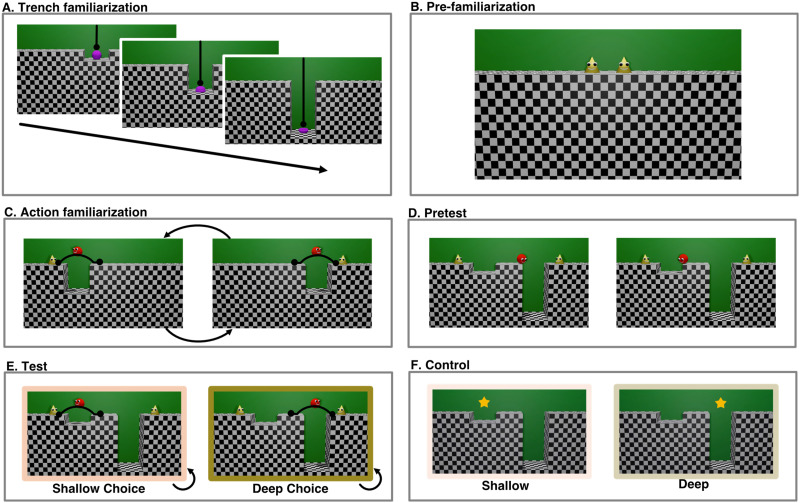
**Trial and event structure of Experiment 3.** Infants first saw (A) a soft ball drop into a shallow, medium, or deep trench, and deform more when it landed in deeper trenches. Next, they saw (B) two identical targets that appeared together at the center of the screen and then moved to the sides of the display and exited the scene. During familiarization that followed (C), infants saw the agent jump over trenches of medium depth to reach these two target characters, on the left and right sides of the display. Then, infants saw test trials in which the agent faced a choice between the two targets, one beyond a shallow trench and the other beyond a deep trench. First, the agent looked down each trench (D) and then alternately chose to jump over each of these obstacles (E). Lastly, during control events (E), an attention-getting star drew infants’ attention above the deep vs shallow trench. Black lines indicate trajectories of motion; circles indicate start- and endpoints of motion; black arrows indicate presentation order (A), and looping (C, E). Across infants, we counterbalanced the side of the deeper trench, the order of the test and control events, and the first sequence of familiarization events.

The displays and counterbalancing structure in Experiment 3 were similar to those from Experiment 2 (Figure 5). Infants saw a trench familiarization trial, a pre-familiarization trial, 6 familiarization trials, a pre-test trial, 2 pairs of test trials, and 1 pair of control trials. All familiarization, test, and control trials included looping events and infant-controlled timing, and the remaining events occurred for fixed durations, as in Experiments 1 and 2.

Relative to Experiment 2, three changes were made to the methods of Experiment 3. The most important change is that infants in Experiment 3 were familiarized to events with no breaking or shattering, or indeed any enduring damage (Figure 5A): a soft ball was dropped from the top of the screen into shallow, medium and deep trenches, deforming to varying degrees as it landed on the trench floor at varying speeds, and then returning to its original shape (3.4 s, 5.2 s, and 6.6 s, with a 0.5 black screen following each event). Corresponding sounds of warping and wobbling accompanied each event. These events, like those of Experiment 2, paired differing outcomes with trenches of different depths, but all of the final outcomes (the ball returning to its original shape) were identical and involved no breakage or observable damage. While prior research has used state changes like breaking and deforming to study infants’ physical, causal, and social understanding (Hauf et al., 2012; Kanakogi et al., 2017; Muentener & Carey, 2010), and although adults may see both object breakage and object deformation as negative, it is unclear whether infants assign negative value to either of these physical state changes. Here we aimed to test whether the results of Experiment 2 are solely attributable to infants having seen deeper trenches paired with the sight and sound of an object breaking.

A second change aimed to clarify the nature of the events by making minor edits to the displays. We provided infants with evidence that there were two targets throughout the study by showing them video clips (Figures 5B), of two identical agents entering (4.0 s) and exiting the scene (7.1s). We also added a video clip during pre-test (Figure 5C) to more clearly convey that the agent could see the depths of the two trenches. In this event, the agent, prior to choosing to jump the shallow versus deep trench, looked down the edge of each trench in turn, making a neutral “Hmmmm” sound (16.2 s total).

Third, we moved the control trials from the start of the experiment to the end (Figure 5E). In Experiment 2, the control trials appeared at the beginning of the experiment, before any events involving the trenches occurred. Thus, it is possible that infants did not closely attend to the trenches during the control trials. By placing the control trials immediately after the test trials, we better equated for infants’ exposure to the rest of the experimental displays across these two sets of trials.

#### Procedure.

Whereas Experiments 1 and 2 were conducted in a quiet, dark room in a lab setting, Experiment 3 was conducted using Zoom video conferencing, in infants’ homes, due to the COVID-19 pandemic, following procedures approved by the Committee on the Use of Human Subjects at Harvard University. We used materials developed by the Stanford Social Learning Lab (Social Learning Lab, 2020) to introduce caregivers to the online testing setup and to ask for verbal consent. Caregivers also provided written consent prior to the study session. Infants sat in a highchair (25 out of 42 participants) or their caregivers’ laps (17/42), depending on caregiver preferences, and watched the displays on a tablet (8/42) or a laptop computer (34/42). We asked caregivers to minimize distractions (pets, people walking by, and mirrors and windows, toys) during the study session.

Before the experiment, infants saw a calibration video where their attention was drawn to the four corners of the screen, as well as the center of the screen. To maximize the quality of the videos seen by infants, we shared our stimuli with caregivers through YouTube playlists, controlled the caregiver’s screen using Zoom’s proprietary remote control feature, and coded infants’ looking times during the study using jHab (Casstevens, 2007). Caregivers rated the quality of the audio and video on a 5-point Likert scale (1 = very poor; 5 = very good), giving high ratings for both (video: *M* = 4.88, *SD* = 0.33; audio: *M* = 4.85, *SD* = 0.36). After the session, we double checked for trial exclusions and generated the final data from the recording of the session video using Datavyu (Datavyu Team, 2014). As before, experimenters only had access to the video feed of infants’ faces (and not the displays) during the experiment, and therefore were unaware of the order of test events. To allow caregivers to attend to potential safety issues at home, we did not ask them to close their eyes, and instead instructed them to refrain from directing their infants’ attention toward or away from the screen. Our full online testing protocol is described in the Supplemental Materials.

#### Data Coding and Analysis.

The data coding and analysis strategy was identical to Experiment 2. Fifty-three out of 504 trials (including familiarization, test, and control trials) were excluded from analysis because of inattentiveness, distractions at home (e.g., pet noises, people walking by), technical issues and coding errors. The proportion of excluded trials (10.52%) was higher than what we observed in the lab in Experiment 2 (6.94%), likely due to distractions in the home environment, the smaller size of the screen displaying the videos at home, and the lower or more variable quality of the video feeds of the infants’ faces (which led to trial mis-timings). As in Experiments 1 and 2, 50% of the test trials were recoded by an additional naive coder (84 of 168 test trials). Interrater reliability was high, *ICC* = 0.96, 95% *CI* [0.93, 0.97].

#### Alternative Hypotheses.

If the results of Experiment 2 are solely explained by infants’ learning to pair breaking and shattering with deeper trenches, then this experiment, which removes these associations, should yield null results. In contrast, if infants enter the experiment with the understanding that falling a greater height leads to more positive cost or negative reward, then the findings of Experiment 3 should replicate those of Experiment 2. Thus, we pre-registered two confirmatory analyses: one comparing infants’ looking times across the two test events, and a second comparing those looking times to infants’ looking preferences during the control events that immediately followed, showing the same physical situation in the presence of an object rather than an agent.

### Results

#### Preregistered Results.

We fully replicated the two key results from Experiment 2. Infants looked longer at test when the agent chose to jump the deeper trench (*M*_*deep*_ = 22.35 s, *SE* = 1.26; *M*_*shallow*_ = 17.55 s, *SE* = 1.00; [0.09, 0.41], *β* = 0.47, *t*(41) = 3.06, *p* = .002, one-tailed, *d* = 0.96, no influential participants). Infants’ looking preferences between the control events and the test events significantly differed from each other ([0.13, 0.75], *β* = 0.74, *t*(105.17) = 2.76, *p* = .007, two-tailed, *d* = 0.54, excluding 1 influential participant).

#### Exploratory Results.

During the control events, infants showed a numerical but non-significant preference for the event in which the inanimate object appeared over the shallower trench (*M*_*deep*_ = 12.09 s, *M*_*shallow*_ = 13.9 s, pooled *SE* = 1.213, [−0.42, 0.07], *β* = −0.17, *t*(64) = −1.39, *p* = .171, two-tailed, *d* = −0.35, excluding 2 influential participants). See Figure 4A.

### Discussion

Experiment 3 fully replicated the key results of Experiment 2: Infants looked longer when an agent chose to jump a deeper trench than a shallower trench, even though both obstacles were equally different in depth from the medium trenches in familiarization, and the agent’s actions were identical across the two events. This pattern of looking differed from when infants’ attention was simply drawn over the deep and shallow trenches. The results of Experiment 3 also confirm that infants did not learn, over the course of the experiment, to associate deeper trenches with worse physical outcomes. Infants saw the deeper trenches as more dangerous even though the object that fell into the trench did not break on impact and returned to its original form, apparently unharmed. It remains possible, however, that infants viewed the temporary deformation of that object as a negative event. To our knowledge, no research to date has tested whether infants see physical state changes in an inanimate object, like breaking or deforming, as intrinsically negative (though see Kanakogi et al., 2013, 2022) for evidence that infants see temporary deformation of an agent as negative).

## EXPERIMENT 4

In Experiment 4, we investigated the developmental origins of the capacity to reason about danger by testing infants under one year of age, using the respective methods of Experiments 1–3. We will reference these samples as Experiment 4, Studies 1, 2, and 3, parallel to Experiments 1, 2, and 3. All 3 studies focused on 10-month-olds because of their previous success in reasoning about the physical costs of actions (Liu et al., 2017).

### Methods

#### Participants.

Our final sample included a grand total of 102 10-month-old infants. Studies 1 and 2 were conducted in the lab, and our final sample included 32 infants in Study 1 (*M* = 10.13 months, range = 9.60–10.63, 15 female; an additional 6 infants tested and excluded from the final sample), and 30 infants in Study 2 (*M* = 9.95 months, range = 8.97–10.47, 17 female; an additional 2 infants tested and excluded). In Study 3, we collected an online sample of 40 infants (*M* = 10.2 months, range = 9.53–11.1, 20 female, an additional 9 infants tested and excluded). In the online sample, infants sat in a highchair (13 out of 40 participants) or their caregivers’ laps (27/40), depending on caregiver preferences, and watched the displays on a tablet (12/40) or a laptop computer (28/40). Caregivers gave high ratings for both the video quality (*M* = 4.88, *SD* = 0.33) and audio quality (*M* = 4.86, *SD* = 0.34). All three of these studies were pre-registered (Study 1: https://osf.io/7k8dt; Study 2: https://osf.io/twsaq; Study 3: https://osf.io/tm9h5).

#### Data Reliability.

As in Experiments 1–3, the reliability of the looking time data in Experiment 4 was high (Study 1: ICC = 0.995, 95% CI [0.991, 0.997]; Study 2: ICC = 0.999, 95% CI [0.998, 0.999]; Study 3: ICC = 0.909, 95% CI [0.859, 0.942])

### Results

#### Inferring Value from Danger (Study 1).

When we tested 10-month-old infants using identical protocols as reported in Experiment 1, these younger infants did not show a statistically significant looking preference between the test events, (*M*_*lower*_ = 19.51 s, *M*_*higher*_ = 19.15 s, [−0.121, 0.301], *β* = 0.168, *B* = 0.09, *SE* = 0.106, *p* = .202, one-tailed, *d* = 0.31, removing 1 influential participant). Comparing the results from Experiment 1 and Study 1, 10- and 13-month-old infants did not significantly differ in their looking preferences in this task, [0.009, 0.4], *β* = −0.202, *B* = −0.115, *SE* = 0.142, *p* = .422, two-tailed, *d* = −0.21, removing 1 influential participant. See Figure S5. Comparing 10-month-old infants’ looking responses in Study 1 to their looking responses to a similar prior experiment that manipulates trench width (Liu et al., 2017, Experiment 3), infants’ responses to the test events did not differ depending on whether trench depth or width was manipulated, [−0.082, 0.568], *β* = 0.289, *B* = 0.243, *SE* = 0.166, *p* = .149, two-tailed, *d* = 0.37, removing 1 influential participant.

#### Avoiding Danger (Studies 2–3).

When we tested 10-month-old infants in identical protocols as in Experiment 2, infants looked longer at test when the agent chose the deeper over the shallower trench (*M*_*deep*_ = 24.97 s, *M*_*shallow*_ = 20.31 s, pooled *SE* = 1.51, [−0.472, −0.047], *β* = −0.386, *B* = −0.26, *SE* = 0.107, *p* = .011, one-tailed *d* = 0.92, removing 1 influential participant). During control events, 10-month-old infants did not show a significant looking preference (*M*_*deep*_ = 12.74 s, *M*_*shallow*_ = 14.68 s, pooled *SE* = 1.97, [−0.174, 0.301], *β* = 0.109, *B* = 0.064, *SE* = 0.119, *p* = .598, two-tailed, *d* = −0.2, excluding 1 influential participant). These two patterns of looking preference did not differ from each other, [−0.728, 0.073], *β* = −0.483, *B* = −0.327, *SE* = 0.205, *p* = .115, two-tailed, *d* = 0.05, no influential participants.

Notably, we did not replicate the results of Study 2 when we ran an additional online sample of infants, using the same methods of Experiment 3: In Study 3, 10-month-old infants did not show a looking preference during the test events (*M*_*deep*_ = 16.03 s, *M*_*shallow*_ = 18.22 s, pooled *SE* = 1.26, [−0.067, 0.339], *β* = 0.251, *B* = 0.136, *SE* = 0.102, *p* = .0965, one-tailed, *d* = 0.43, excluding 1 influential participant), or the control events (*M*_*deep*_ = 12.74 s, *M*_*shallow*_ = 12.30 s, pooled *SE* = 1.24, [−0.213, 0.269], *β* = 0.05, *B* = 0.028, *SE* = 0.121, *p* = .818, two-tailed *d* = −0.13, no influential participants), and their looking preferences did not differ across the two phases of the experiment, [−0.28, 0.37], *β* = 0.08, *B* = 0.05, *SE* = 0.17, *p* = .78, two-tailed, *d* = 0.05, no influential observations.

Pooling data across older and younger infants tested in Experiments 2–3, and in Studies 2–3 of Experiment 4, we found a marginal 3-way interaction between cliff depth (shallow vs deep), phase of experiment (control vs test), and age group (infants younger than 1 y vs older than 1 y), [−0.001, 0.699], *β* = 0.349, *B* = 0.349, *SE* = 0.179, *p* = .053, two-tailed, *d* = 0.2, excluding 1 influential participant. This interaction appeared to be driven by differences in younger and older infants’ responses to the test events: we found a significant interaction between age group (younger vs older than 1 y) and cliff depth (shallow vs deep) for the test events ([0.053, 0.453], *β* = 0.253, *B* = 0.253, *SE* = 0.103, *p* = .015, two-tailed, *d* = 0.36, excluding 1 influential participant), but not the control events ([−0.362, 0.119], *β* = −0.121, *B* = −0.121, *SE* = 0.123, *p* =. 326, two-tailed, *d* = −0.18, excluding 2 influential participants). Thus, in a large sample (*N* = 142), infants younger and infants older than 1 year of age differed in their pattern of looking responses to events where agents choose more vs less dangerous actions, but did not differ when their attention was simply drawn to the physical trenches where these actions occurred. See Figure S5.

## GENERAL DISCUSSION

When we reason about the minds and actions of other people, we consider not only what they wanted and did, but also the time and energy that they devoted to their actions. Recent work suggests that even young children and infants use tradeoffs between reward and cost to understand the actions of other people. Formally, this ability is captured by models of forward planning, that use known information about other agents’ utility functions to predict their actions, and inverse planning, that allow observers to infer other agents’ utility functions given their overt actions (Baker et al., 2009, 2017; Jara-Ettinger et al., 2015, 2016; Liu et al., 2017). In this paper, we asked whether infants can assign negative utility to physical actions that always succeed, but that *could* have failed and resulted in bad outcomes. We operationalized danger as the height that another agent could fall from, following classic work on depth perception and motor development (Adolph, 2000; Gibson & Walk, 1960). Across three experiments, one-year-old infants used relative danger to predict and explain an agent’s actions. They expected the agent to minimize danger, looking longer when the agent chose a more perilous action when a safer action was available (Experiment 2–3). Moreover, infants used the danger that an agent faced to infer the relative value of the goal state that the action achieved (Experiment 1). When tested on these same tasks, infants younger than 1 year of age either did not succeed (Experiment 4, Study 1), or did not succeed consistently (Experiment 4, Studies 2–3). Pooling data across all the studies here reported, a reliable age effect emerged. These findings suggest that the ability to assess the danger of an action, or the ability to use danger in calculating the cost of a successful action and the reward value of the goal state that the action achieves, either emerges or strengthens around the start of the second year.

Although our highly controlled stimuli limited our ability to test whether infants understand the danger underlying actions in more naturalistic settings, they allowed us to study infants’ responses to one variable—the height that an agent could fall from—while controlling for all other aspects of that agent’s actions. Whereas past research tested infants’ sensitivity to the costs of actions that also varied along perceptual features, like path length and vertical displacement, and involved physical costs of different magnitudes (Liu et al., 2017), our experiments isolate and test for infants’ sensitivity to the differing danger of two actions that were identical with respect to physical and perceptual variables. The consistent effects of relative danger on one-year-old infants’ looking patterns provide evidence that infants represent danger as a variable in an integrated calculus, trading off positive rewards and negative costs. These findings suggest that our understanding of people’s actions, even in infancy, is based not only on what someone chooses to do, but also what would have happened if a chosen, successful action had gone awry.

Before discussing further implications of our findings, we would like to address one alternative interpretation of the results from Experiments 2 and 3. We interpreted infants’ longer looking when the agent chose to jump the deeper cliff as evidence that infants expected an outcome (the agent choosing to jump the shallower trench) that did not occur. But in Experiments 2–3, infants could have looked longer when the agent chose to jump over the deeper trench not because it was surprising, but because infants more vigilantly monitored this jump. Under this interpretation, infants still assigned negative utility to potential falls from greater heights but did not have an expectation about which action the agent would choose. This account predicts that infants would also look longer at, and be less likely to look away from, jumps over deeper trenches during the familiarization events from Experiment 1. This prediction was not supported by the data: Infants were equally attentive to all familiarization events from Experiment 1, regardless of whether the agent jumped or backed away, and regardless of how deep the trench was (see SM for details). It was only when agents had a *choice* between two trenches, during the test events of Experiments 2–3, that infants showed a looking preference. Altogether, we believe that our results support the interpretation of looking during the test events as reflecting expectations about action outcomes under conditions of varying danger, rather than lower-level preferences for deeper trenches, learning during the experiment to associate deeper trenches with the sight and sound of objects breaking and shattering, or heightened vigilance when agents jumped over deeper trenches.

The early-developing sensitivity to the negative utility of dangerous actions leaves open three key questions. First, what is the computational basis for one-year-old infants’ sensitivity to danger? Past work on action understanding conceived of the utility of other agents as composed of two variables: the reward of goal states, *R*(*S*), and the cost of actions, *C*(*A*) (Equation 1) (Jara-Ettinger et al., 2016):

$$U\left(A,S\right)=R\left(S\right)-C\left(A\right)$$ (1)

Our findings suggest either of two possible extensions to Equation 1 to capture the notion of danger. The first extension assigns an additional negative value to the action, which expresses the danger of an action, *D*(*A*), without explicitly representing either the possible states that could result from the action (i.e., achieving the goal, falling down the trench), or the probability that they will occur:

$$U\left(A,S\right)=R\left(S\right)-C\left(A\right)-D\left(A\right)$$ (2)

Under this conception of danger, jumping over deep trenches carries negative utility defined over the surrounding context of the action, as does physical exertion, so a reward must be greater to justify both efforts and dangers. In other words, dangerous actions are dangerous in and of themselves, as some inherent feature. Running at full speed, next to a cliff, is directly, inherently, and immediately dangerous.

But there is a different extension to Equation 1, which suggests a richer interpretation of our findings. Infants may represent the set of possible states, *S*, that could result from an action, *A*, each occurring with probability *P*(*S*_*i*_|*A*). Each state is associated with a separate reward, *R*(*S*_*i*_): Reaching a goal state carries positive utility, while transitioning to a state of injury carries negative utility.

$$U\left(A,S\right)=\sum_{S_{i}\in S}P\left(S_{i}|A\right)R\left(S_{i}\right)-C\left(A\right)$$ (3)

This notion of danger is closer to the notion of expected utility. It takes into account the rewards (positive and negative) of possible states (observed and unobserved) that could result from an action, and the likelihood of transitioning to each state. Running full speed next to a cliff, under this definition, is dangerous because of the potential consequences of tripping and falling.

Conceiving of danger in the second way requires more than perceiving the actions that occurred and the states they led to: One must also posit states of the world that did not occur, but could have resulted from a given action (counterfactuals), or states of the world that could occur but have not yet occurred (hypotheticals). The development of counterfactual and hypothetical reasoning remains controversial (e.g., Carey et al., 2020; Harris et al., 1996; Kominsky et al., 2021; Nyhout & Ganea, 2018; Rafetseder & Perner, 2010). However, we note that a commitment to inverse planning as a model of intuitive psychology requires a commitment at least to hypothetical representations, because the forward model of rational planning must assign utilities across multiple actions and states (e.g., choosing the apple vs the banana, jumping the trench vs declining to jump) in order to calculate the likelihood of each of these actions. If forward and inverse planning indeed guides infants’ action understanding, then the difference between Equations 2 and 3 boils down to an ability to nest hypotheticals – to assign multiple states to a single action, and then hold in mind these states nested within actions in order to compare utilities across actions. Future research might shed light on the nature and development of this understanding, by leveraging behavioral and neural correlates of simulation that are sensitive enough to distinguish between these hypotheses (e.g., Gerstenberg et al., 2017; Schuck & Niv, 2019).

A second open question is whether infants represent danger in terms of bodily states, mental states, or both. That is, do 13-month-old infants view the deeper trench as harboring greater injury to an agent who fails to clear it, as eliciting negative emotion in an agent as it jumps (Gjata et al., 2022; Ruba et al., 2019; Skerry & Spelke, 2014), or as requiring greater attention or care (Scott & Baillargeon, 2013)? Further research exploring children’s attributions of emotion to agents, and testing for expectations of other people’s vigilance during dangerous situations, might shed further light on this question.

A third question concerns the development of infants’ sensitivity to danger. How do infants learn to see successful actions, performed with no negative consequences, as dangerous? We have shown, when pooling data across experiments, that younger and older infants differ in their looking behavior towards these stimuli. Even at 10 months of age (the mean age of the infants in Experiment 4), research provides evidence that infants have the physical understanding required to infer that the body of an agent could fall without a supporting surface (Needham & Baillargeon, 1993), that jumping comes at a physical cost (Gergely & Csibra, 2003), that agents tend to minimize this cost (Liu & Spelke, 2017), and that the choice to incur a large cost for a goal is evidence that the agent assigns high reward to that goal (Liu et al., 2017). We propose that even with these capacities, there are still challenges associated with success in the current study, including recognizing that an agent could fall, even though it never does, and inferring that a fall to a greater depth is worse than a fall to a lesser depth, especially given that the actions of agents in these varying contexts were physically identical.

What accounts for the possible development over the ages of 10–15 months? It may not be coincidental that these months span the age at which infants become capable of our species-specific, upright locomotion. In the current study, 10-month-old infants, who, on average, have not yet begun to stand or walk independently, do not show consistent responses to the dangerous actions of other agents. Given that specific experience with walking and crawling predicts infant’s behavior when they are placed in similar situations as those featured in these stimuli (Kretch & Adolph, 2013), It is possible that the experiences that accompany motor development provide infants with insight into other people’s action plans. Infants may learn from their own experience navigating with and without physical support, or from the reactions of their caregivers when they find themselves in these situations, that there is something negative about acting under lack of support. It is unclear whether 10-month-old infants cannot appreciate the danger in the actions shown in these events, or whether they would succeed in similar tasks with lower task demands. Future studies could explore the emergence of infants’ attributions of danger and of the developmental changes that underlie it, including developmental changes in motor abilities, working memory, action planning, and counterfactual and hypothetical reasoning.

### Conclusion

The present findings provide evidence that our early commonsense understanding of other people’s actions includes representations of danger–in the current research, the height from which an agent could fall, even if this outcome never occurs. Twelve to 15-month-old infants expect others to maximize the utility of their actions in an integrated calculus where danger trades off systematically against reward. Specifically, infants expect others to minimize the danger of their actions, and they infer the value of an agent’s goal from the danger of the action that the agent undertook to achieve it. Infants show this expectation when they are presented with a novel agent whose actions never ended in failure, based only on the physical situations in which those actions occurred. Finally, these findings suggest that infants use forward planning models, describing how other agents choose actions on the basis of their utilities (defined over reward, danger, and effort), and invert these models to reason about the variables that bear on these choices. For one-year-old infants, “danger” is one of the abstract variables that infants may attribute to agents who plan and act under uncertainty.

## ACKNOWLEDGMENTS

We thank the families who volunteered to participate, members of the Harvard Lab for Developmental Studies for helpful discussion, the Cambridge Writing Group for writing feedback, Linette Kunin for help with creating stimuli, Jane Hu for help with data collection, and Melyssa Almeida, Cameron Calderwood, Caitlin Connolly, Linette Kunin, Laura Lee, Yuman Li, and Vanessa Kudrnova for help with data coding. Funding: NSF CCF-1231216, Siegel Foundation Award S4881, DARPA CW3013552, NSF GRFP DGE-1144152 (to SL), NIH F32HD103363 (to SL).

## AUTHOR CONTRIBUTIONS

SL, BP, and MG carried out the experiments. SL analyzed the data and wrote the first draft of the manuscript, and TU, JT, and ES helped provide critical feedback on the paper. All authors helped to design the research.

## OPEN PRACTICES STATEMENT

All experiments reported in this paper were formally pre-registered. All stimuli, data, code, and pre-registrations of this paper are open access at https://osf.io/kz7br/.
