# Supplementary material for: Dangerous Ground: One-Year-Old Infants are Sensitive to Peril in Other Agents’ Action Plans
Source: Open Mind (Camb). 2022 Oct 30;6:211–31. doi: 10.1162/opmi_a_00063 (PMC9692054; doi:10.1162/opmi_a_00063)
Supplement: Supplementary file 1 [file opmi-06-211-s001.pdf]

Supplemental Materials for the manuscript

“Dangerous ground: One-year-old infants are sensitive to peril in other agents’ action plans”

For data and code required to reproduce these figures and results, see https://osf.io/kz7br/.
Please direct questions to Shari Liu, shariliu@jhu.edu.

Contents:
Supplemental Figures S1-S6
Supplemental Tables S1-S5
Supplemental Results
1. Results including influential observations
2. Exploring order effects in Experiment 1
3. Analyses over looking behavior during familiarization
4. Pilot study on adults, comparing manipulations of width vs depth
5. Online testing materials

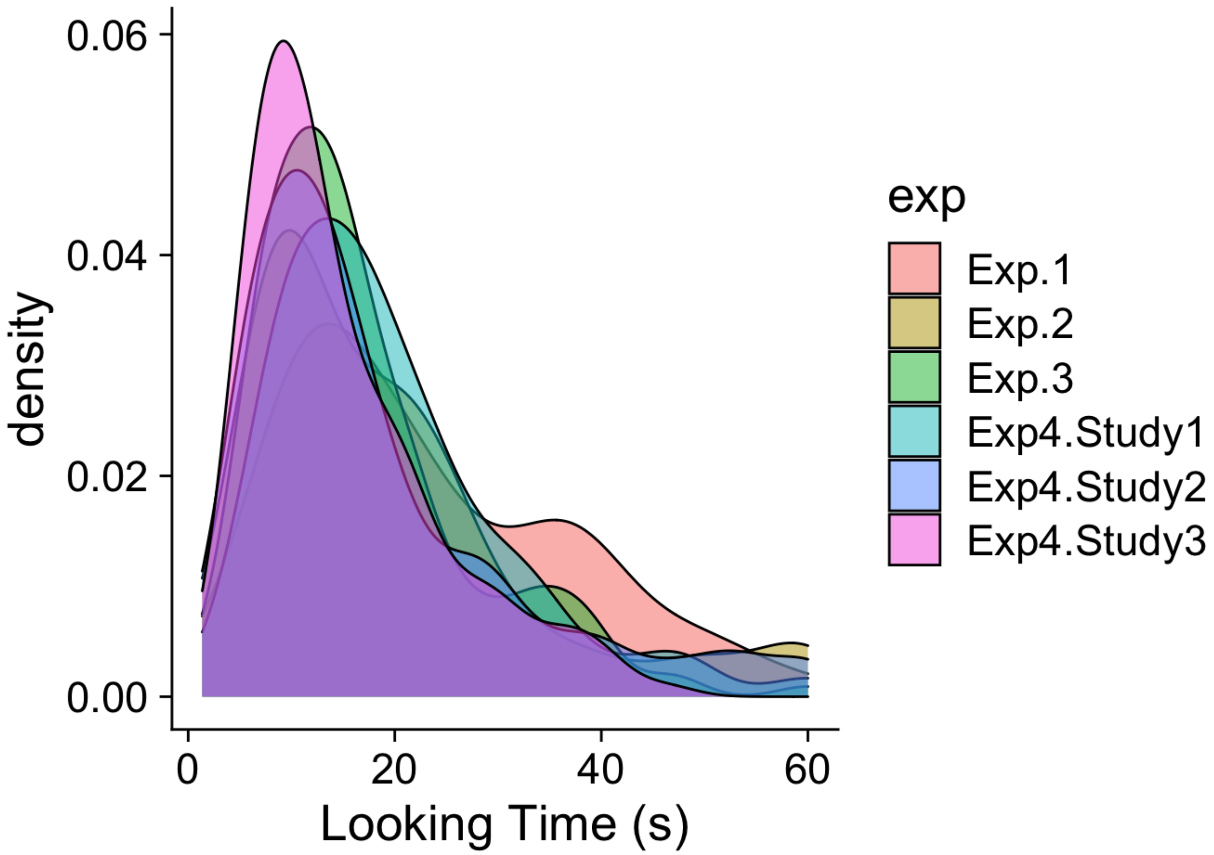

Figure S1. Density plot of looking times during test for Experiment 1 and from test events and control events for Experiments 2-4. Maximum-likelihood fitting revealed that the lognormal distribution (log likelihood=-2456.26) provides a better fit to these data than the normal distribution (log likelihood=-2624.35).

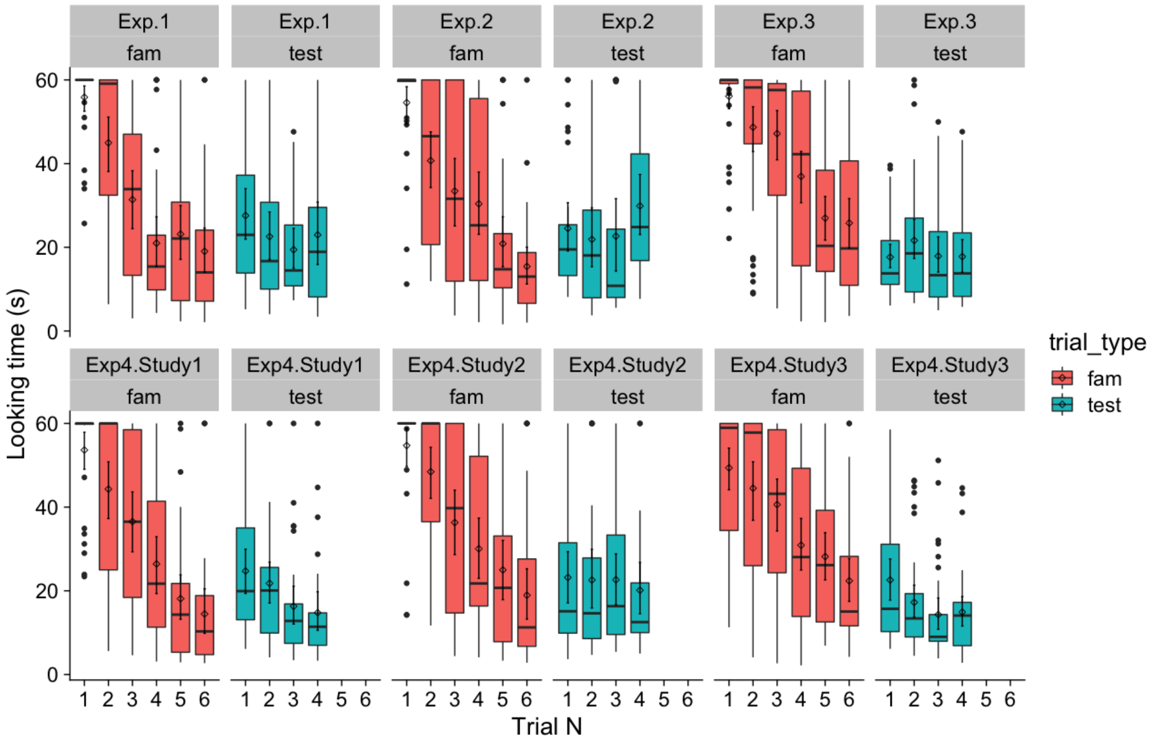

Figure S2. Boxplots of looking times during familiarization and test across Experiments 1-4 (total N=206; Exp.1b-3b indicate Studies 1-3 in Experiment 4). Error bars represent bootstrapped 95% confidence intervals around the mean.

Supplemental Results

1. Including influential observations

Below, we report the results from our pre-registered analyses including all observations, rather than excluding influential observations.

Experiment 1. Infants looked longer at test when the agent, at test, chose the deeper trench over the shallower trench ([0.02,0.41], *ß* = 0.35, $t(31) = 2.18$, $p = .037$, two-tailed). These findings accord with those reported in the main text and support the interpretation that infants expected the agent to choose the goal for which it was willing to jump deeper trenches.

Experiment 2. Infants looked longer at test when the agent, at test, chose the deeper trench over the shallower trench ([0.07,0.51], *ß* = -1.07, $t(29) = 2.59$, p = .008, one-tailed). During control events, 13-month-old infants preferred to look at the shallow trench ([-0.37,-0.02], *ß* = -0.34, $t(27.3) = -2.20$, $p = .036$, two-tailed). Their looking preferences significantly differed across the two phases of the experiment, [0.11,0.88], *ß* = 0.75, $t(84.74) = 2.52$, p = .013, two-tailed). These findings accord with those reported in the main text and support the interpretation that infants expected the agent to take the less dangerous action and therefore showed a greater looking preference for the test event than for the control event presenting events over the deeper trench.

Experiment 3. Infants looked longer at test when the agent chose to jump over the deeper trench ([0.09,0.41], *ß* = -1.05, $t(41) = 3.06$, $p = .002$, one-tailed). During control events, infants did not show a looking preference for either event ([-0.41,0.17], *ß* = -0.20, $t(68) = -0.82$, $p = .418$, two-tailed). Their looking preferences significantly differed across the test and control trials ([0.04,0.70], *ß* = 0.60, $t(108.49) = 2.18$, $p = .032$, two-tailed). This finding fully replicates the two key findings from Experiment 2 and accords with the findings reported in the main text.

2. Order effects in Experiment 1 (reviewer-requested exploratory analysis)

Infants' looking preferences at test did not vary depending on which sequence of events (low to high danger vs high to low danger) they were randomly assigned to watch in the first familiarization trial ([-0.20,0.55], $ß$ = 0.29, $t(30)$ = 0.93, p = .362, two-tailed). All infants saw both trial orders for 3 familiarization trials each.

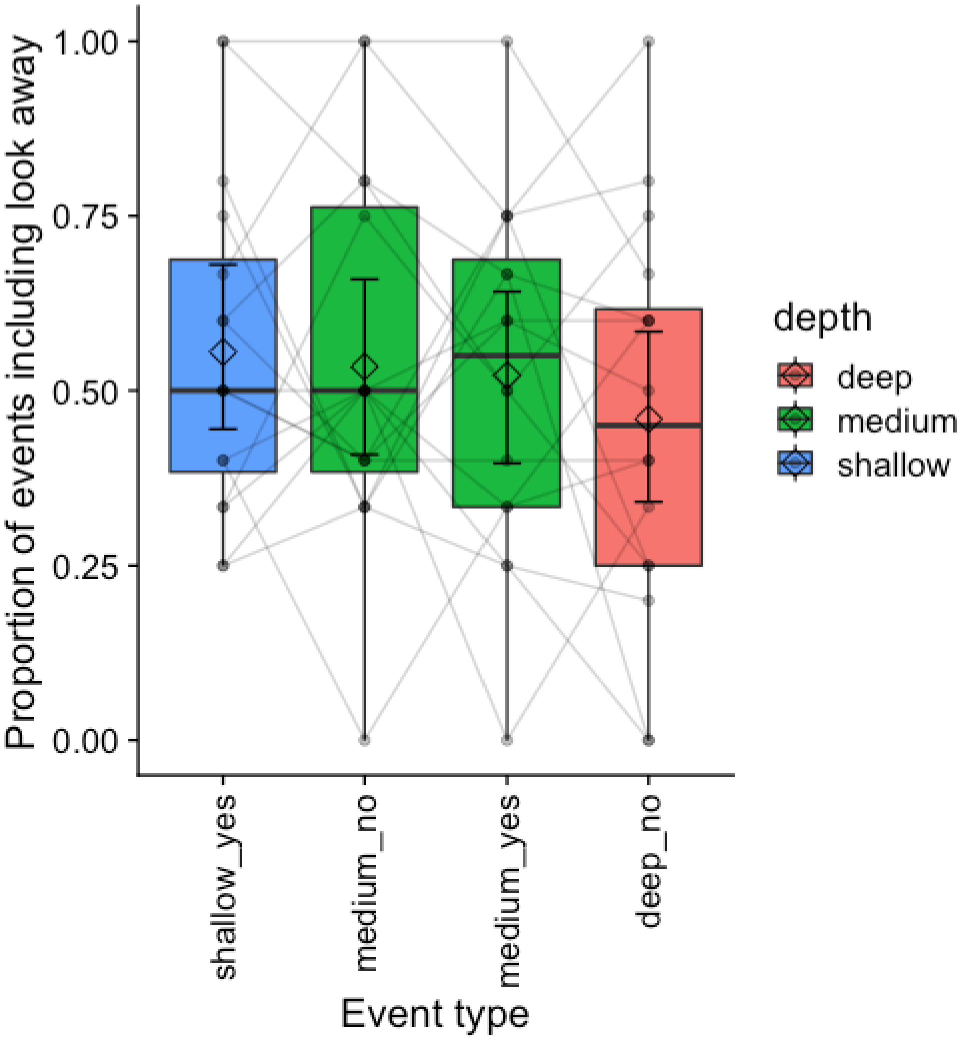

Figure S3. Proportion of events during which infants glanced away from the screen, relative to how many times infants saw each event. Data come from a random subset of infants in Experiment 1 (N=16 out of 32 total infants), with observations grouped by infant (points connected by grey lines). Error bars represent bootstrapped 95% confidence intervals around the mean. Infants look away from the screen with roughly equal probabilities across the 4 event types.

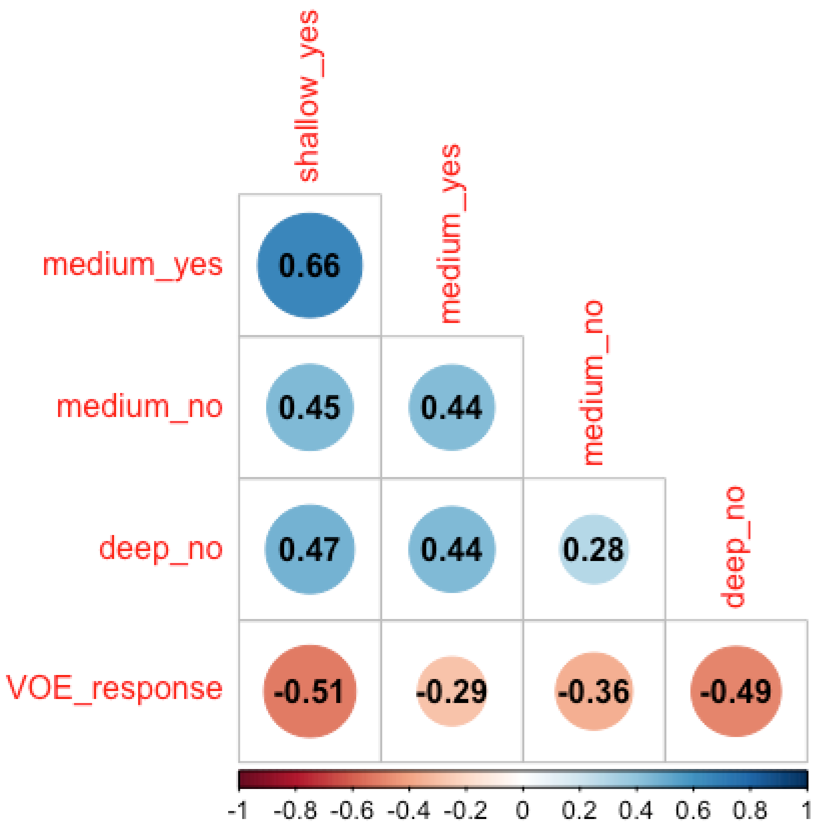

Figure S4. Correlation plot relating infants' likelihood of looking away from each of the 4 familiarization events (proportion of events including a look away) to one another, and to infants' violation of expectation response (unexpected - expected) at test. Values indicate Pearson's correlations. Descriptively, the more infants looked away from the events, the smaller VOE response they showed at test.

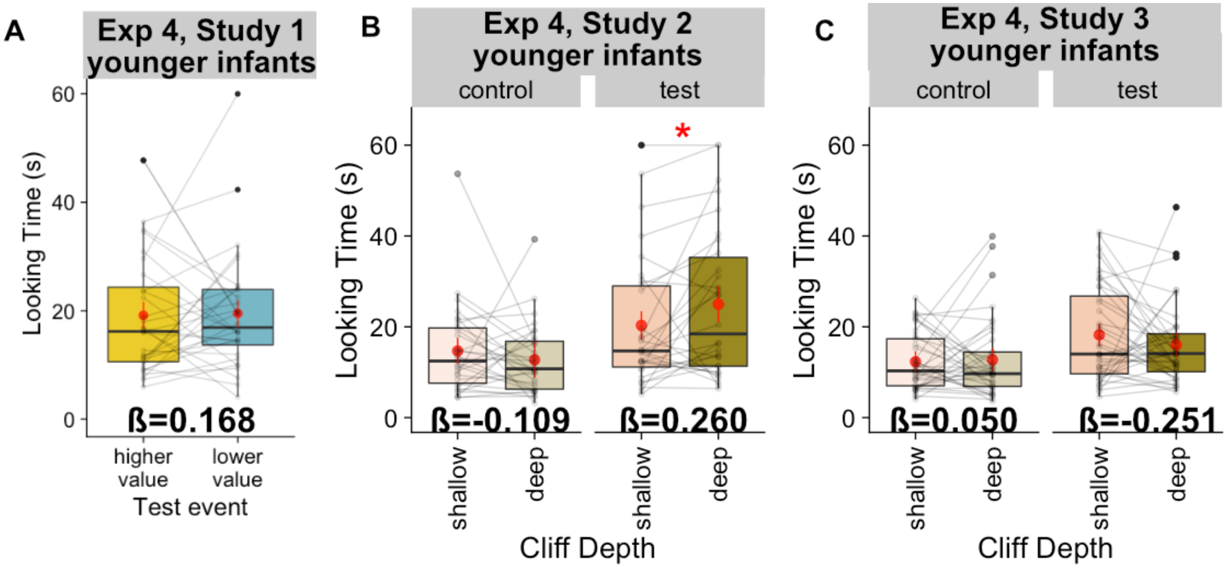

Figure S5. Looking time towards test and control events in Experiment 4, Studies 1-3. Red error bars around means indicate within-subjects 95% confidence intervals. Pairs of points indicate data from a single participant. Horizontal bars within boxes indicate medians, and boxes indicate the middle 2 quartiles of data. Beta coefficients (ß) list effect sizes in standard deviations. P * < .05, ** <.01, *** <.001 (pre-registered as one-tailed for test events due to directional prediction, two-tailed in all other cases).

3. Attention during each familiarization event in Experiment 1 (reviewer-requested exploratory analysis)

In Experiment 1, infants, on average, looked longer when the agent jumped deeper trenches for one goal than another, and then chose the other goal later at test. One question is how infants used the information in each of the 4 familiarization events, presented in a looping sequence over 6 familiarization trials, in order to draw this inference. Rather than comparing the relative acceptances and refusals of the agent across 3 different levels of peril (shallow, medium, and deep trenches), one alternative hypothesis is that infants selectively attended when the agent accepted and refused the same obstacle (medium trench) for the two goals, and used this ‘go-no-go’ heuristic to infer that the agent prefers the goal it jumped for, over the goal it refused to jump for. Furthermore, in Experiments 2-3, infants looked longer when the agent jumped the deeper trench. Does this longer looking reflect an expectation that was violated, or heightened vigilance when the agent chose an action that could end in worse outcomes?

Under the alternative hypothesis for Experiment 1, infants should be less likely to glance away from events involving medium trenches (vs the other events), and those who looked away less (i.e. attended more) to the medium trench events should have exhibited larger violation-of-expectation effects at test. Under the alternative hypothesis for Experiments 2-3, infants in Experiment 1 should be less likely to look away from events involving deeper trenches than shallow ones. To test these predictions, naive coders chose a random 50% of videos from Experiment 1 and annotated the onset and offset times of each iteration of each event in each familiarization loop, ignoring interleaved blank screens, and then annotated the onset and offset of infants’ attention to each event iteration. In the plots and following analyses, the events are named *shallow_yes* and *medium_yes* when the agent willingly jumped a shallow or medium trench, and *medium_no* and *deep_no* when the agent refused to jump a medium or deep trench. For each infant, we calculated the number of each kind of event they saw. Then, we calculated the proportion of those events that infants looked away from. If an infant looked away from the screen for any portion of the event, we marked that event as one where they looked away. Otherwise, we marked that event as one where they looked the entire duration. For example, if an infant saw 5 *deep_no* events and glanced away from the screen for 1 of them, this produced a score of 0.2 for that event type, for that infant. We then averaged these proportions within infants across all 4 event types, to produce 4 different proportion glance-away scores per infant. These scores are plotted in Figure S3, are related to each other, and to infants’ looking preferences at test, in Figure S4. The model summary is shown in Table S1, and data and analysis scripts are available at https://osf.io/kz7br/.

Overall, infants were equally likely to glance away from the screen (vs attend for the entire duration) during the 4 events. See Table S1 for results of the linear mixed effects model (lmer formula: `prop.glancedoff ~ videoclip + (1|subj)`). Thus, infants did not attend selectively to the events where they had the opportunity to compare the agent’s acceptance and refusal of the medium trench towards the two goals (alternative explanation for Experiment 1). They also did not more vigilantly monitor events that occurred near the deep trench (alternative explanation for Experiments 2-3). Instead, they were equally likely to glance away from all 4 types of events.

Table S1. Infants’ probability of glancing away from the 4 video clips from familiarization in Experiment 1

		Probability of looking away to each video clip			
Predictors	***Estimates***	***95% CI***	***Statistic***	***p***	***df***
(Intercept) [deep_no]	0.92	0.89 – 0.95	56.12	**<0.001**	58.00
videoclip [medium_no]	-0.02	-0.06 – 0.02	-1.11	0.268	58.00
videoclip [medium_yes]	-0.02	-0.06 – 0.02	-0.80	0.421	58.00
videoclip [shallow_yes]	-0.01	-0.05 – 0.03	-0.36	0.717	58.00

We then tested the second prediction: that infants who glanced away from the medium trench events (i.e. those who missed the critical information for a go-no-go strategy) would also show smaller violation-of-expectation responses at test. To do this, we calculated infants’ looking preference at test (average duration looking when the agent chose the less valued goal, minus average duration looking when the agent chose the more valued goal), and asked whether variability in infants’ looking behavior towards each of the 4 events predicted variability in these looking preferences. We found that infants’ tendency to glance away from the events involving medium trenches, or towards any of the 4 events, did not predict the magnitude of their violation-of-expectation response. See Table S2 for full results (lm formula: `delta.look ~ shallow_yes + medium_no + medium_yes + deep_no`).

Together, these findings suggest that infants did not selectively attend to the videos with the same trench depth during familiarization in Experiment 1 (or selectively glance away from the other events), and that their looking towards these videos did not predict stronger inferences

about which goal was more valuable. Furthermore, they did not more vigilantly monitor the agent when it moved closer to deeper trenches. Instead, they attended roughly equally to all 4 events. Therefore, it appears unlikely that infants as a group used a “go-no-go” heuristic on the agent’s actions over the medium trenches in order to infer which the agent preferred in Experiment 1, and that infants in subsequent experiments looked longer at the agent’s actions over the deeper trenches solely out of vigilance. To be clear, we are not suggesting that infants could *never* use a go-no-go strategy, or more carefully monitor dangerous-looking events. Instead we are suggesting that these alternative explanations do not appear to account for the results in the main text of this paper.

Table S2. Infants’ violation of expectation responses at test, as predicted by their tendency to glance away from the 4 video clips from familiarization in Experiment 1. Dependent and independent variables were z-scored prior to entry into the model.

	Violation of expectation response (unexpected - expected)				
Predictors	***Estimates***	***95% CI***	***Statistic***	***p***	***df***
(Intercept)	0.00	-0.49 – 0.49	0.00	1.000	11.00
shallow_yes	-0.01	-0.56 – 0.55	-0.03	0.973	11.00
medium_no	0.12	-0.44 – 0.68	0.48	0.643	11.00
medium_yes	0.33	-0.31 – 0.97	1.13	0.284	11.00
deep_no	0.37	-0.19 – 0.94	1.45	0.176	11.00

4. Pilot study on adults, comparing manipulations of depth and width

Participants. 19 adult participants were recruited to participate via Amazon Mechanical Turk after providing informed consent.

Experiment overview. Research protocols were approved by the Harvard University Institutional Review Board. Adults saw an agent poised on the edge of a trench of varying depth and width (4 units per axis), and an object of varying color and shape that stood beyond the other edge of the trench. For each image, adults were asked to imagine that the agent attempts to jump across the trench to reach the object on the other side, and respond to the following questions using a sliding scale:

1. **Force:** How much force will this agent need to expend in order to successfully perform the action? (0 = This agent will need to expend very little force; 100 = This agent will need to expend a great amount of force)
2. **Probability:** What do you think is likely to happen? (0 = The agent will definitely fail; 100 = The agent will definitely succeed)
3. **Tired:** Imagine that the agent successfully jumps across the cliff. How tired will they be afterwards? (0 - This agent will not be tired at all; 100 - This agent will be extremely tired)

Subjects were also asked other sliding scale questions, about the emotional state of the agent, which are not the focus of the current research and were used to motivate a different published paper (Gjata et al., 2022, *Cognitive Science*). Of the 16 possible stimuli (4 widths x 4 depths), participants saw 8 stimuli randomly selected from this set, and responded to these three questions in a fixed order per participant, but randomized across experiments. These responses are shown in Figure S6.

Results. For each of the 3 dependent measures, we asked whether the manipulation of depth and width, as well as their interaction, explained variability in people's responses. We fit one linear mixed effects model per dependent measure, using the formula `` `response ~ depth * width + (1|subj)` ``. For all three dependent measures, we found that the manipulation of depth significantly predicted people's judgments of how much force is required to jump across the trench, the probability that this jump will be successful, and how tired the agent will feel afterwards. The wider the trench an agent faced, people judged that a jump across that trench would require more force (*B*=20.24, 95% *CI* [13.12, 26.36], *p*<.001, two-tailed), be less likely to be successful (*B*=-20.58, 95% *CI* [-27.92, -13.25], *p*<.001, two-tailed), and make the agent more tired (*B*=16.10, 95% *CI* [8.99, 23.21], *p*<.001, two-tailed). Manipulating trench depth by the

same amount did not impact people’s responses on these any measures (all ps > .25), at least in this small pilot sample, and the impact of manipulating depth and width did not interact. The model summaries are shown in Tables S3-S5, and data and analysis scripts are available at https://osf.io/kz7br/.

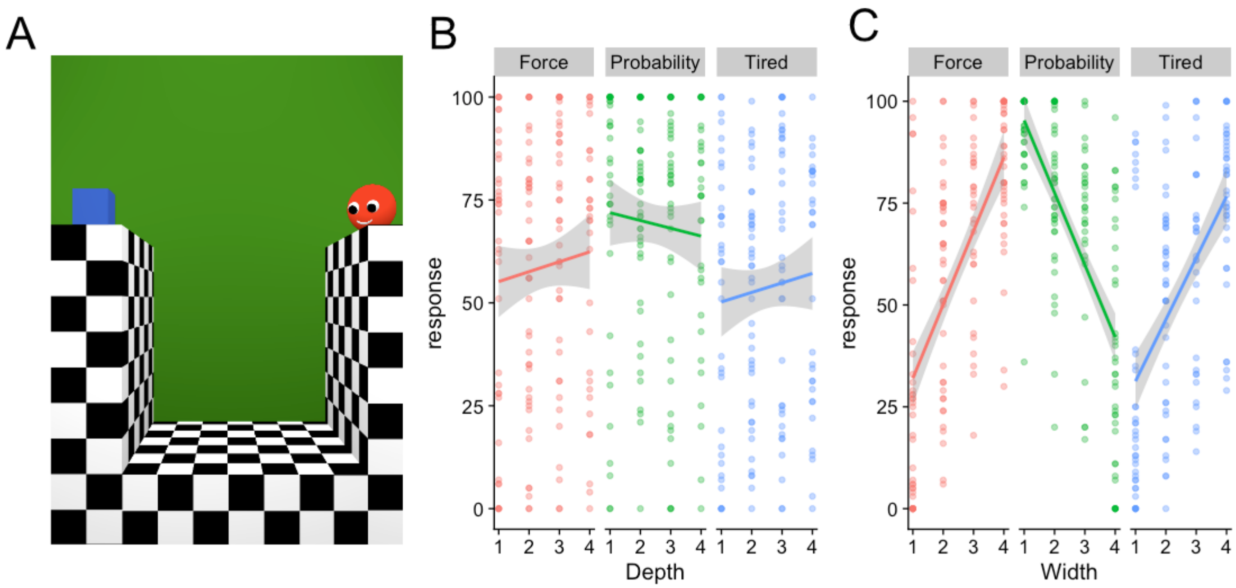

Figure S6. (A) Example of stimuli shown to participants. (B-C) People’s judgments about the degree of force required, the probability of success, and how tired the agent would be, as a function of the depth (B) or width (C) of the trench the agent jumps over. Raw data are plotted as individual points, and a line of best fit with a 95% confidence interval was produced using `geom_smooth(method = “lm”)`.

Table S3. The effect of manipulating trench depth and width on judgments of force required to jump. (0 = This agent will need to expend very little force; 100 = This agent will need to expend a great amount of force)

	Force judgments				
Predictors	***Estimates***	***CI***	***t***	***p***	***df***
(Intercept)	2.94	-17.70 – 23.58	0.28	0.779	146.00
Depth	4.39	-3.17 – 11.94	1.15	0.253	146.00
**Width**	**20.24**	**13.12 – 27.36**	**5.62**	**<0.001**	**146.00**
Depth * Width	-0.83	-3.55 – 1.88	-0.61	0.544	146.00
Random Effects					
σ^2	380.49				
N subj	19				
Observations	152				
Marginal R^2 / Conditional R^2	0.416 / 0.645				

Table S4. The effect of manipulating trench depth and width on judgments of probability of success. (0 = The agent will definitely fail; 100 = The agent will definitely succeed)

	Probability judgments				
Predictors	***Estimates***	***CI***	***Statistic***	***p***	***df***
(Intercept)	122.53	102.30 – 142.76	11.97	<0.001	146.00
Depth	-4.31	-12.05 – 3.43	-1.10	0.273	146.00
**Width**	**-20.58**	**-27.92 – -13.25**	**-5.55**	**<0.001**	**146.00**
Depth * Width	1.32	-1.46 – 4.10	0.94	0.351	146.00
Random Effects					
σ^2	419.88				
N subj	19				
Observations	152				
Marginal R^2 / Conditional R^2	0.455 / 0.521				

Table S5. The effect of manipulating trench depth and width on judgments of the agent’s energy after jumping across. (0 - This agent will not be tired at all; 100 - This agent will be extremely tired)

	Energy judgments				
Predictors	***Estimates***	***CI***	***Statistic***	***p***	***df***
(Intercept)	7.34	-13.69 – 28.36	0.69	0.491	146.00
Depth	3.60	-3.95 – 11.15	0.94	0.348	146.00
**Width**	**16.10**	**8.99 – 23.21**	**4.47**	**<0.001**	**146.00**
Depth * Width	-0.40	-3.11 – 2.31	-0.29	0.771	146.00
Random Effects					
σ^2	377.98				
N subj	19				
Observations	152				
Marginal R^2 / Conditional R^2	0.307 / 0.628				

5. Online testing materials

Instructions for parents, sent over email

Dear [Caregiver Name],

We are emailing you in order to remind you that we have [Infant’s Name] scheduled to participate in a study with us [Date and Time]. The Zoom link for your appointment is: [Zoom link] and the password required to enter is [password].

Here are a few things to consider for your appointment:

1. For the first 5 minutes of our Zoom call, we will be setting up (e.g. testing out video/audio to make sure everything sounds and looks good). Your baby does not need to be present for this part of the call.
2. For the actual study, we will ask you to sit them in a high chair (our preferred setup) or in your lap, while they watch our videos on a desktop or laptop computer. Think about what you and your baby would prefer, and which room would be best suited for the study. Ideally, the location would be quiet, and relatively distraction-free.
3. During the 5-10 minutes where your baby will watch our videos, we'll ask you to not direct your baby's attention towards or away from the screen. For those minutes, we'll also ask, if possible, to keep pets, siblings, and other household members occupied and (ideally) in a separate room. (We understand that this is not always possible!) We will also ask you to keep toys and other attractive objects out of the baby's immediate reach or view.

Please let us know if you have any additional questions before your appointment, otherwise we look forward to seeing you both soon!

Best,

[Researcher Name]

Researcher Checklist

Before the experiment (at least 15 min ahead of time)

- Check written consent form, and send a reminder to caregivers if they still need to send. Archive consent form.
- Log session info (participant ID, condition, etc).
- Disable all computer notifications (Slack, email, etc)
- Set up jHab.
- Set up introduction slides for the testing session.
- Choose the right playlist for the session. Double-check that it's correct.
- Log into the password-protected Zoom room a few minutes early (recording should automatically start)

During the experiment

- Introduce yourself and the study. Be sure to ask if caregivers have questions. Remind them that the baby does not need to be present for the first 5 minutes.
- Ask for verbal consent from caregivers
- Guide caregivers through the following steps for video setup
  - Turn off computer/iPad notifications, e.g. do not disturb
  - Open YouTube/Vimeo playlist of stimuli, press pause.
  - Zoom -> Share Screen -> Desktop 1. Check "share computer sound"
  - Go back to YouTube, and go to full screen.
  - Zoom -> Minimize view of self and the experimenter(s).
  - Zoom -> More … > Hide floating meeting controls
  - Zoom -> Grant remote control to experimenter - might need to go into accessibility options to grant permission
  - Verify that both parties can hear the videos, and that caregiver cannot see anything except the video.
- Guide caregivers through the following steps for baby setup
  - Move all toys and attractive objects out of view and reach
  - If possible, minimize pets and other people walking into and out of the room
  - Seat baby in a high chair (preferred) or in lap, with the laptop/computer/iPad on a table surface
  - Place the laptop/computer/iPad as close as possible to the baby, without the baby being able to reach forward and touch it.
  - Check for screen glare
  - Tilt the camera such that you can see the baby's face, and their torso
  - If the baby is seated in a high chair, seat the caregiver beside and behind the infant. Do not ask caregivers to close their eyes (in case something happens at home that they need to attend to), but do ask caregivers to be neutral, and to not direct their baby's attention.
- Running the study
  - Make sure views of stimuli are covered before starting the study.

  - Turn off your own video and audio. Before you do this, ask caregivers if they have questions, and remind them that they can pause or stop the study at any time for any reason.
  - If the baby seems uncomfortable or fussy, pause in between trials to touch base with caregivers.
- After the study
  - Go through debriefing slides with caregivers
  - Record device information, and video-audio ratings
  - Ask for suggestions and feedback - caregivers often have really good ideas!
  - Make sure caregivers know how to contact you if they have further questions or comments

After the experiment

- Archive consent form
- Archive video recording, and generate picture-in-picture archival video
- Send caregivers gift card
- Update FileMaker log
- Log all other columns of session info (device, audio/video ratings, etc).
- Code the video (do this as soon as possible after the session). Double-check any trials that may have been miscoded, included a distraction, etc
